# High striped hyena density suggests coexistence with humans in an agricultural landscape, Rajasthan

**DOI:** 10.1371/journal.pone.0266832

**Published:** 2022-05-04

**Authors:** Debashish Panda, Subham Mohanty, Tanuj Suryan, Puneet Pandey, Hang Lee, Randeep Singh

**Affiliations:** 1 Amity Institute of Forestry and Wildlife, Amity University Uttar Pradesh, Gautam Buddha Nagar, Uttar Pradesh, India; 2 Department of Zoology, North Campus, Delhi University, New Delhi, India; 3 Conservation Genome Resource Bank for Korean Wildlife (CGRB), Research Institute for Veterinary Science and College of Veterinary Medicine, Seoul National University, Seoul, Republic of Korea; Zoological Survey of India, INDIA

## Abstract

Understanding the mechanism of coexistence, where carnivores adapt to humans and vice versa in the shared landscape, is a key determinant of long-term carnivore conservation but is yet to be comprehensively examined. We explored the coexistence mechanism of striped hyena (*Hyaena hyaena)* and humans in the shared landscape of Sawai Mansingh Wildlife Sanctuary (SMS WLS), Rajasthan, from November 2019 to March 2021. We used data derived from motion sensors-based surveys, satellite remote sensing images, and household questionnaires to understand socio-ecological, environmental and anthropogenic factors facilitating hyena persistence in the shared landscape. The high density (12 individuals/100 km^2^) striped hyena in the landscape revealed the coexistence with humans. Being scavengers, they get subsidised food sources and are perceived as low-risk species by humans. Striped hyena minimised temporal activity during the daytime when human activity peaked. However, the highest activity overlap was observed in the agricultural area (Δ1 = 0.39), and likely depicts the high activity due to agricultural practices. While the human settlement was positively associated with the detection of hyenas, the probability of striped hyena captures increased with decreasing distance from human settlement, possibly influenced by high carcass availability, providing the easiest food resources to striped hyena, and allowing them to coexist with humans. This study demonstrates the coexistence of hyenas and humans in the shared landscape supported by mutual benefits, where hyenas benefit from anthropogenic food from scavenging, while humans benefit from waste removal and the non-lethal nature hyenas.

## Introduction

Expanding the footprint of humans has modified ~ 70% of Earth’s land surface [1, 2], causing not only the loss of carnivore habitat but also affecting the animal movement and global species recovery efforts [3]. Because of landscape modification, an increasing number of large carnivore species have been forced to inhabit with humans in modified landscapes (hereafter shared landscape) [4], which likely have escalated the human-carnivore interactions [5]. The extent to which humans co-adapt with the carnivore in the shared landscape is the key to the success of coexistence [6]. However, carnivores pose several real and perceived threats to humans (i.e., economic loss and deaths) living in their vicinity [7]. These threats directly lead to retaliatory killings and local extinction of large carnivores due to intolerant behaviours by humans, threatening carnivore conservation efforts [8]. However, the behavioural adaptation of large carnivores (i.e., reduced home range in human-dominated areas and increased nocturnal activity) [9], their socio-ecological importance [10, 11], and human tolerance are key mechanisms facilitating coexistence in shared landscapes [12–14].

Despite the several national and international efforts to protect carnivores globally, the populations continue to decline in response to increasing human populations and political instability [15]. World current population projections indicate further expansion of human footprint globally when ~2.0 billion people are expected to add to the current human population by 2050 [16]. For the long-term persistence and strengthening of the conservation efforts of carnivores in the Anthropocene, an understanding of (i) how carnivores adapt and use the matrix (remnant of natural vegetation) in shared landscapes [17, 18], and (ii) how carnivores render benefits to humans’ well-being, are the key determinants [19]. For example, spotted hyenas (*Crocuta crocuta*) in northern Ethiopia are valued and tolerated for their service of removal of carcasses of livestock and reducing the risk of disease [20]. In the Himalayan region, because of the cultural and religious beliefs, the Tibetan Buddhist monasteries protect snow leopards (*Panthera uncia*) and their habitats, although snow leopard heavily depredates on their livestock [21]. After the eradication and recent recolonisation of large carnivores in certain regions of Europe and the USA, changes in human behaviour and tolerance towards carnivores have been observed [7, 15].

India is home to 23% of global carnivore species, wherein the carnivores share space with a population of 1.3 billion people in multiuse landscapes (e.g., forest, agroforests, scrublands, barren lands, grasslands) [22]. Among these, ~4% forest is protected, while ~19% are unprotected forest cover of the whole country’s land area [23], which has been used by carnivores for different purposes, including foraging, dispersal, and reproduction [24]. Carnivore generally adopted behavioural mechanism can lead to coexistence in shared landscapes via (i) spatial avoidance of human-dominated areas [5] (ii) overlap in the same space with humans but temporally avoid humans, e.g., nocturnality [25]. However, shared landscapes are recently recognised as potential habitats for many wildlife species of conservation interest [15, 26]. However, for the long-term persistence of carnivores in shared landscapes, it is essential to identify the ecological and anthropogenic factors that facilitate human-carnivore coexistence [27].

In this paper, we focused on striped hyena (*Hyaena hyaena)*, a species listed as Near Threatened by the International Union for Conservation of Nature (IUCN) Red List [28], as a model species to understand the coexistence pattern with humans. The striped hyena is a large-bodied, asocial, and solitary carnivore [29]. It exhibits nocturnal activity [29] and is widely distributed in India’s arid and semiarid landscapes [30]. It is a ‘facultative scavenger’ adapted to coexist with humans and mostly scavenges on domestic and wild ungulate carcasses [30]. We studied habitat use and interactions between the striped hyena and humans in the shared landscape (dominated by agro-pastoral activities) of Sawai Mansingh Wildlife Sanctuary (SMS WLS), Rajasthan, India. Based on our prior knowledge of the ecology of the species and previous research in the landscape [31], we used data derived from motion sensors-based surveys and satellite remote sensing images and household questionnaires to understand how ecological and anthropogenic factors facilitate their persistence in the shared landscape. We aimed to understand the major drivers of coexistence between the striped hyena and human in the shared landscape in the following hypotheses; (i) being a scavenger; hyena does not attack livestock and humans; hence humans tolerate striped hyenas as they clean the organic waste generated by humans and reduce disease risk [32]. Hence, we predict a higher density of striped hyenas in the shared landscape, (ii) behavioral adjustments (i.e., nocturnality) of striped hyena tend to minimise human interactions. Hyenas in the landscape use the same space as humans but temporally avoid humans and (iii) spatial partitioning of spatial avoidance of humans via spatial use of the landscape by which hyenas reduce interaction with humans. In this study, we direct a comprehensive view towards the ecological attributes of striped hyenas, which are helpful to managers and conservationists to accurately determine parameters influencing striped hyena’s presence for optimising investment in the management of resources.

## Material and methods

### Ethics statement

This study was conducted after getting permission from Rajasthan Forest Department (letter no- F 19(11) permission/cwlw/2017/1678). We followed all guidelines for animal care and scientific research ethics.

### Study area

**Fig 1:**
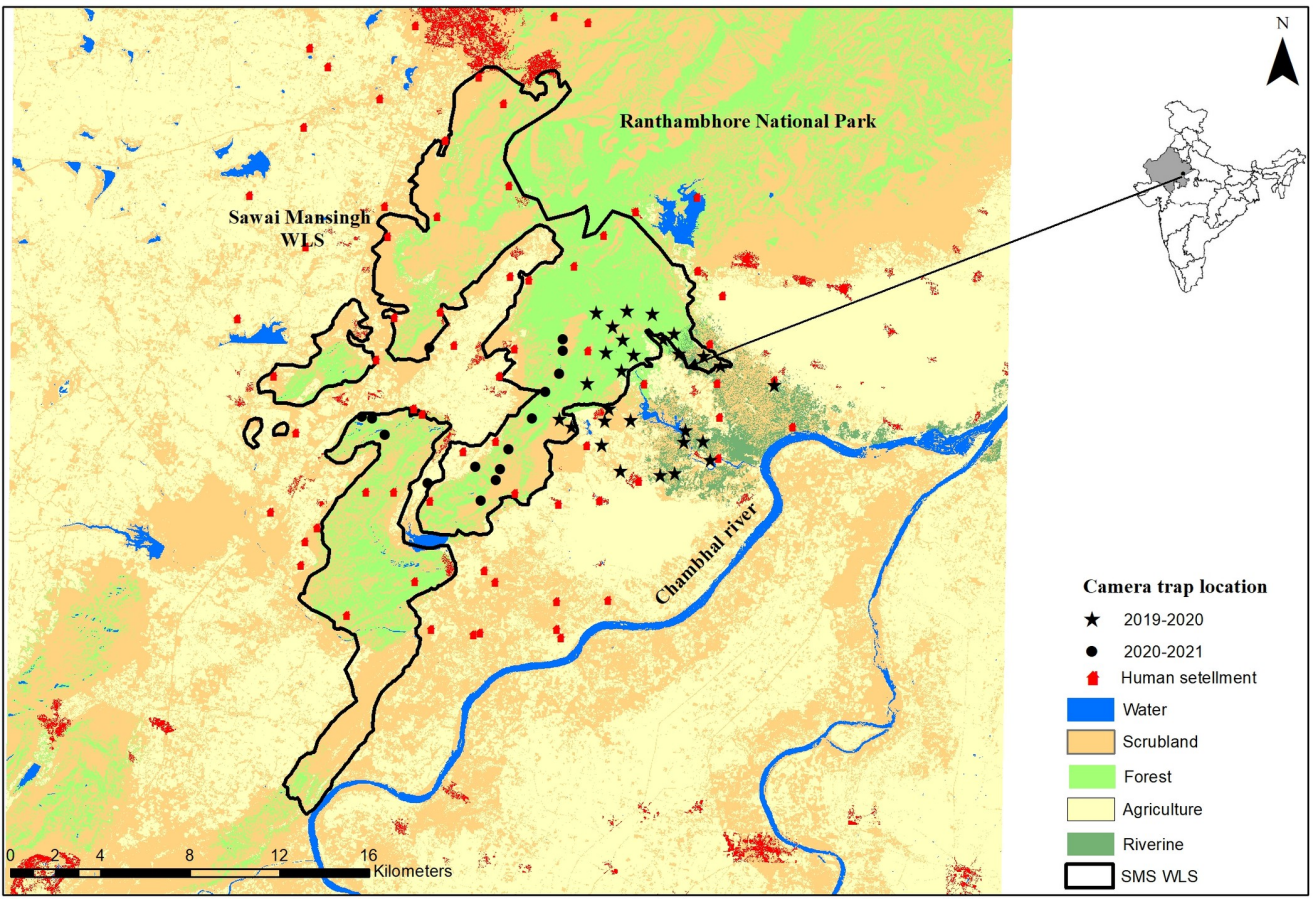
Location map of the study area and camera trap location in Sawai Mansingh Wildlife Sanctuary Rajasthan from November 2019 to March 2021.

We conducted this study inside and buffer area of Sawai Mansingh Wildlife Sanctuary (SMS WLS), Rajasthan, India (Fig 1). The SMS WLS is a part of the tiger conservation and management unit of the Ranthambhore Tiger Reserve [33]. The total area of SMS WLS is 127.6 km^2^, while another adjacent forested area is Qualji area 7.58 km^2^ and another forested area 132.96 km^2^ [34]. The entire Ranthambhore landscape forms a transition zone between the true desert and seasonally wet peninsular India [35]. The area falls in the 4B semiarid zone and Gujarat-Rajwara biotic province [36]. The region’s average annual rainfall is 800 mm, of which 500 mm falls in the monsoon season. The temperatures can be ≤ 2°C in January and ≥ 47°C in May [31]. The landscape is undulating and dominated by humans; there are 75 villages within a 5 km buffer of SMS WLS with more than 104261 people inhabiting in and around [37]. The rolling landscape mosaic is interspersed with forest, scrublands, grasslands, riverine areas, and agricultural lands [31]. The residents are mostly engaged in agriculture, livestock farming, cutting grass, grazing livestock, lopping trees, and mining (illegally) to supplement household incomes. All the villages are primarily dependent on agriculture for their livelihood, and their economy is supplemented by animal husbandry. They have numerous cows, buffaloes, and goats but very few herds of sheep and camel. The villagers tend to graze their animals in the fallow agricultural lands and the village commons during the lean periods of the year, viz., January to June. However, the villagers enter the peripheral forest area to graze their animals throughout the year. The area is dominated by northern tropical, dry, deciduous, and thorny forest [38]. The forests are mainly of edaphic climax and belong to the subgroup 5B- Northern Tropical Dry Deciduous forests and subgroup 6B -DS1-Zizyphus scrub [38]. The degradation stages are DS1-Dry deciduous scrub and SS4 -Dry Grasslands [38]. The vegetation was representative of a typical dry deciduous dhok forest (*Anogeissus pendula*). Apart from dhok, the species commonly found are kadaya (*Sterculia urens*), salai (*Boswellia serrata*), raunj (*Acacia leucophloea*), amaltas (*Cassia fistula*), Palash (*Butea monosperma*), tendu (*Diospyros melanoxylon*), gurjan (*Lannea coromandelica*), and Jamun (*Syzygium cumini*). Apart from humans’ landscape is shared with the striped hyena, tiger (*Panthera tigris*), leopard (*Panthera pardus*), sloth bear (*Melursus ursinus*), jackal (*Canis aureus*), fox (*Vulpes bengalensis*), and the wild ungulates, including sambar (*Rusa unicolor*), chital (*Axis axis*), nilgai (*Boselaphus tragocamelus*), chinkara (*Gazella gazelle*) and wild boar (*Sus scrofa*).

### Hyena detection data

We obtained striped hyena detection data via a camera-trap survey in different land use categories in the landscape. The landscape was characterised into five different land-use classes (forest habitat, scrubland, agriculture area, riverine habitat, and water) based on recent satellite imagery (S1 File in S1 Text, S1 Table in S1 Text). We conducted a reconnaissance survey initially to define the extent of the hyena distribution in different land-use classes. Then, we overlaid the grid cell network of 1 × 1 km^2^ over the distribution area of hyena in the area. Forty-three sampling sites for the winters of both years (Table 1) were selected randomly from the grid of 52 cells, with forest = 14, scrubland = 06, agriculture = 11, riverine habitat = 12 located landscapes (Fig 1). We used ten camera trap devices for sampling belonging to Cuddeback C1 type; WI, USA digital camera, (20.0 megapixel) enabled with white flash. Camera traps were enabled to take three subsequent photos bursts every time the sensor was triggered. We set the sensitivity of the camera minimum value and set traps 6-7m apart from the animal trail, so each camera had sufficient time to detect the animal and take full-frame pictures.

**Table 1:** Summary of camera trapping effort at the study site of Sawai Mansingh Wildlife Sanctuary, Rajasthan during 2019–2020 and 2020–2021.

Sampling duration	Sampled area (Km^2^)	No. of trapping station	Sampling days	Trap Night	Photo Captures			Identified hyena	Density(D)±SE/100 km^2^	g0±SE	Sigma(σ)±SE (in Km)
					Total	Left	Right				
November 2019- March 2020	38.76 Km^2^	28	34	952	125	64	61	14	12±0.03	0.04±0.01	1.46±0.18
November 2020-February 2021	36.33 km^2^	15	34	510	143	73	70	14	11±0.03	0.05±0.01	1.55±0.23

The single-camera traps were installed per sampling site on either side of the road. To capture hyena’s natural behaviour, no lures or baits were used. According to Coordinated Universal Time (UTC), camera time was set as the time standard approaches and time and date stamps imprinted to each image when the camera was triggered. The sampling was conducted during winter seasons only from Nov 4 2019, to Mar 18, 2020, and Nov 21 2020, to Mar 7 2021. Camera trap stations were spaced 1 km apart from nearby traps (average trap distance = 924m). The camera traps were installed 45–60 cm above ground and operational for 34 days per sampling site. Both the animals and humans share the same space; hence, to avoid cameras having been stolen or damaged by locals, 21 camera traps (close to human habitation) were deployed at evening 18:00 and removed in the morning 07:00 daily. Despite these four-camera trap units being stolen, the data were not considered in the analysis. Instead, we collected the variables within a 200-m radius around each camera trap site. This was considered the area over which localised conditions may influence species detectability. The minimum convex polygon for season 1 and season 2 covered an area of 38.8 km^2^ and 36.3 km^2^.

### Density estimation

**Fig 2:**
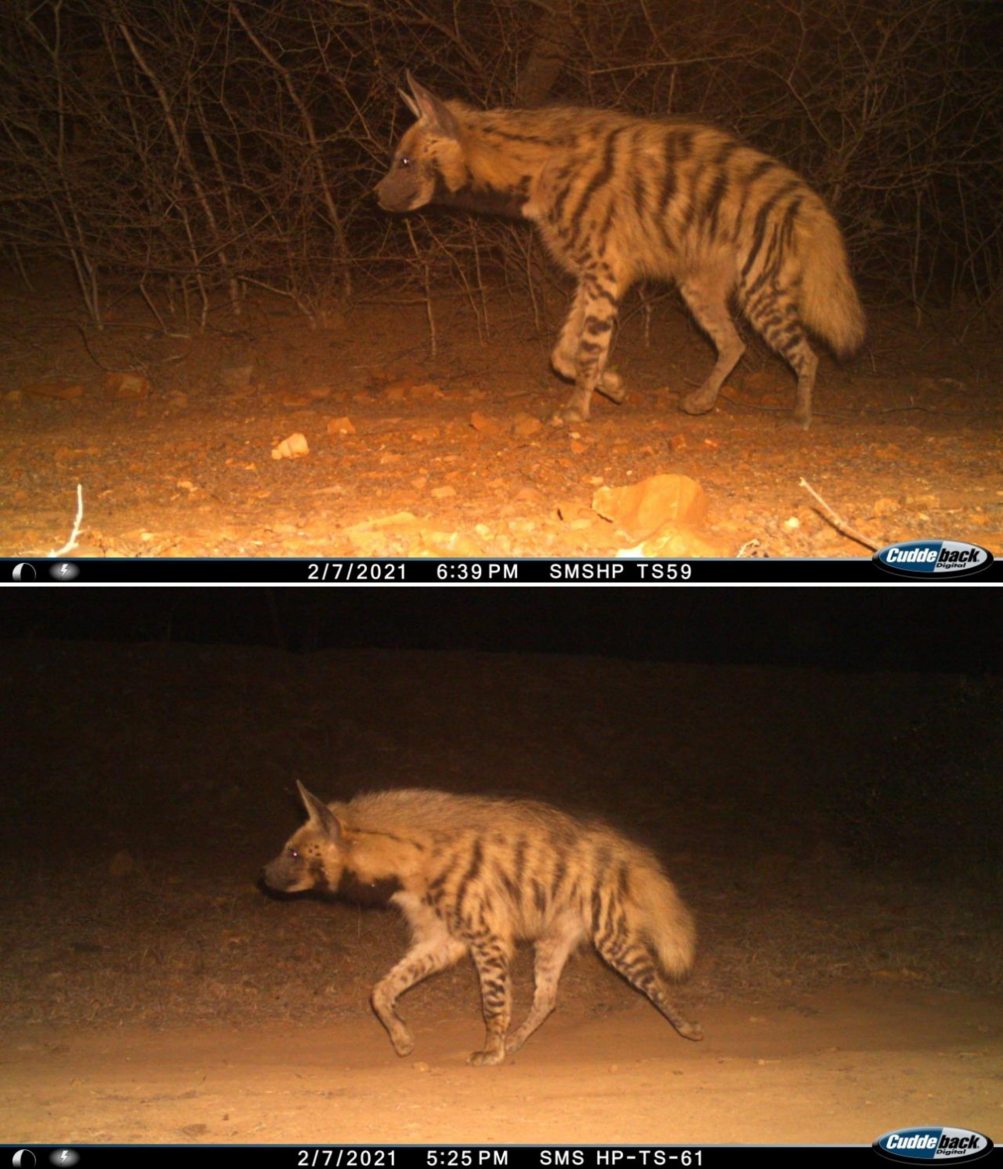
Photo-capture of striped hyena in Sawai Mansingh Wildlife Sanctuary, Rajasthan.

As the number of photo captures of striped hyenas was higher in left flanks, we considered it to estimate the density. Individual striped hyenas were identified from photographs obtained using the camera traps by visually examining the markings on the pelage of the hind limbs, forelimbs, and forequarters (Fig 2) [31]. Photographs in which hyenas were individually identified were assigned unique identification numbers, and the specific trap location, sampling period, date, and time of capture were recorded. We constructed a capture history of striped hyenas in SECR data format for analysis for each sampling session that considered a continuous 34-days sampling occasion. Using the camera trapping data, we followed the spatial explicit capture-recapture (SECR) approach to obtain maximum likelihood density estimates for striped hyenas [39]. The likelihood SECR models were implemented package SECR V. 4.4.8 in the R and DENSITY 5.0.3 [40, 41] (www.Otago.ac.NZ/density). The detection probability of each individual was modelled using the spatial detection function [42] and was explained by two parameters (one-night detection probability at the centre of an individual’s home range, [g_o_] and a function of the scale of animal movements [σ]; [42]. We used a half-normal detection function because it seemed appropriate for mark-recapture data from large carnivores. We evaluated the log-likelihood function by integrating the Poisson distribution of the home range centres by adding a buffer of 10,000 m around the trapping grids (this distance was chosen to ensure that no individual outside of the buffered regions had any probability of being photographed by the camera trap during the survey [43].

### Influence of human activity on striped hyena nocturnality

We examined the diel activity pattern of hyenas and humans in different land use categories (i.e., forest, agriculture, riverine, and scrubland) using the date and time imprint on camera trap images. We considered only independent captures taken from different stations or at least 30 min apart from the same station or depicted unambiguously different individuals in the same station [44, 45]. The association between the hyena and humans’ activity was compared using the Chi-Square test in a different land-use category using Cramer’s V strength of association [46]. The effect size was calculated in the R package ‘effect size’ [47]. The value of association ranges from 0 (no association) to 1 (perfect association). The activity pattern and overlapping coefficient (Δ) was calculated using a non-parametric kernel density estimation method [48] for hyenas and humans in different land-use categories using the ‘overlap’ package [49] in program R v.4.0.2 [50]. The value of overlapping coefficient (Δ) ranges from 0 [no overlap] to 1 [complete overlap] [48]. Recommended by [49], Dhat 4 (Δ4) coefficient of overlap was used for a larger sample size that was more than 75 (>75), and Dhat 1 (Δ1) was used for a smaller sample size that was less than 75 (<75). We used both Δ1 and Δ4 depending on the sample size. We obtained a 95% confidence interval for activity overlap using 10000 bootstrapping iterations. After that, we calculated the activity pattern of a striped hyena at each terrain category using a non-parametric kernel density estimation method [48] in the ’overlap’ package [49] using program R v.4.0.2 [50].

### Spatial use of the landscape by hyena

To assess the effects of landscape and anthropological variables on the detection of hyenas at camera trap stations, we used a Generalised Linear Model (GLM) because it fits the count data well [51]. We used seven environmental variables at every camera trap within the grid, i.e., distance from the human settlement (m), aspects (degrees), slope (degrees), scrubland (%), forest cover (%), and riverine habitat (%) and distance from water (m) (S1 File in S1 Text). A 200m buffer was laid using the “buffer” feature of “Proximity” under “Analysis tools” of ArcMap v.10.2.2 (Esri 2014) to calculate the habitat parameters at each sampling point. We also calculated the Relative abundance Indices (RAI) for humans per site as covariates. RAI was calculated as RAI = E/TNx100, where E is the number of events (photo-captures), and TN is the total number of trap nights [52]. We used the RAI of a hyena (i.e., detection rate) per camera trap station as a response variable, while landscape and anthropogenic variables as a predictor variable. The multicollinearity among variables was examined using IBM SPSS Statistics (ver. 21.0; SPSS Inc., Chicago, IL., USA, variables with VIF (variance inflation factor) <3 were included in the analysis [53]. VIF ranged from 1.3 to 2.8 for all variables (S2 Table in S1 Text). Therefore, all variables were retained in the modelling. We used Poisson distribution with log link function [54]. The list of all possible models was created to examine the relationship between our response variable and predicted variables. We considered the final Model with ΔAIC < 2 using the ‘dredge’ function of package ‘MuMIn’ in program R [55]. Model selection was based on Akaike’s information criterion and Akaike weights [56]. We averaged the parameter coefficients of all models with a cumulative Akaike weight > 0.9 [57, 58]. All analysis was performed in program R v.4.0.2 [50]—data analysis codes used in program R and data given in S1 and S2 Appendices.

### People’s perception of hyenas

We interviewed 200 random people selected from our study area. Respondents were questioned about their occupation, types of crops, conflicting species, hyena’s role in the ecosystem, conflict with the hyena, and attitude towards striped hyena (S2 File in S1 Text). We also asked about livestock mortality rate, mortality reasons, and the process of livestock dumping after death. Furthermore, the livestock number was collected from livestock census data collected by the government for each village in the year 2019–2020 (S3 Table in S1 Text).

## Results

### Striped hyena density

**Fig 3:**
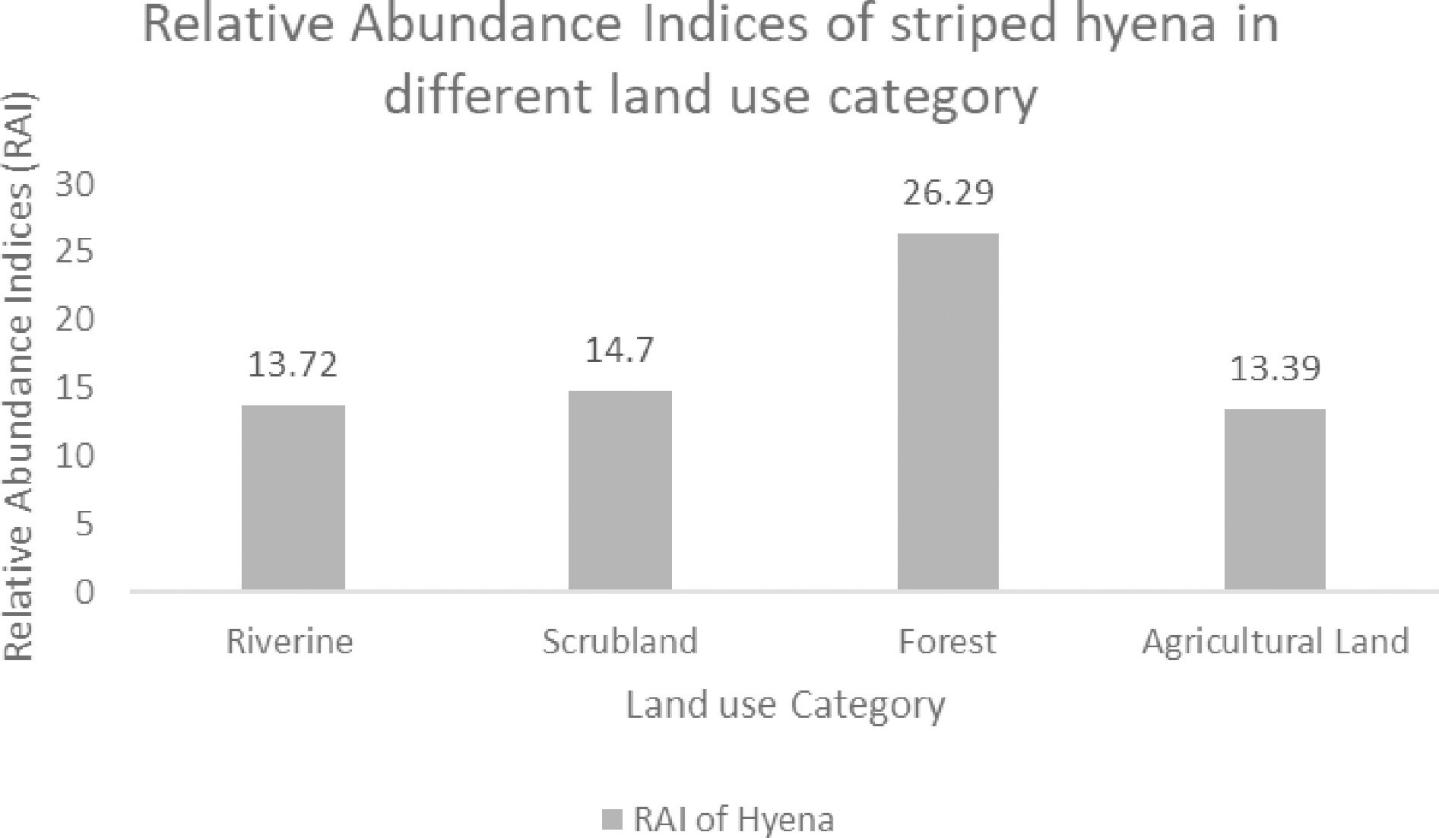
Relative abundance indices (RAI) of hyena in different land-use categories in human dominated landscape of Sawai Mansingh Wildlife Sanctuary, Rajasthan.

A total camera trapping effort of 1462 days captured 28 unique striped hyena individuals spanning two years (Table 1). The number of individual striped hyena captures did not differ between years, although the highest number was captured during the winter season of 2020. The RAI of hyenas and humans was calculated at 18 captures/100 trap days and 139/100 trap days consequently (S4 Table in S1 Text). The RAI of striped hyena was recorded higher in the forest followed by scrubland, riverine, agricultural land, while the human activity was recorded higher in the agricultural area followed by riverine, forest and scrubland (Fig 3). The presence of striped hyenas was least associated with humans in the landscape (r = 0.40, p = <0.05). We recorded the highest striped hyena density for both years in the landscape. The hyena density for each season was estimated at 12±0.03 individuals/100 km^2^ and 11±0.03 individuals/100 km^2^, respectively. However, the animal movements from the centre of the home range (σ) for were 1.46 (SE = 0.18) km and 1.55 (SE = 0.23) km. Consequently.

### Influence of human activity on striped hyena nocturnality

**Fig 4:**
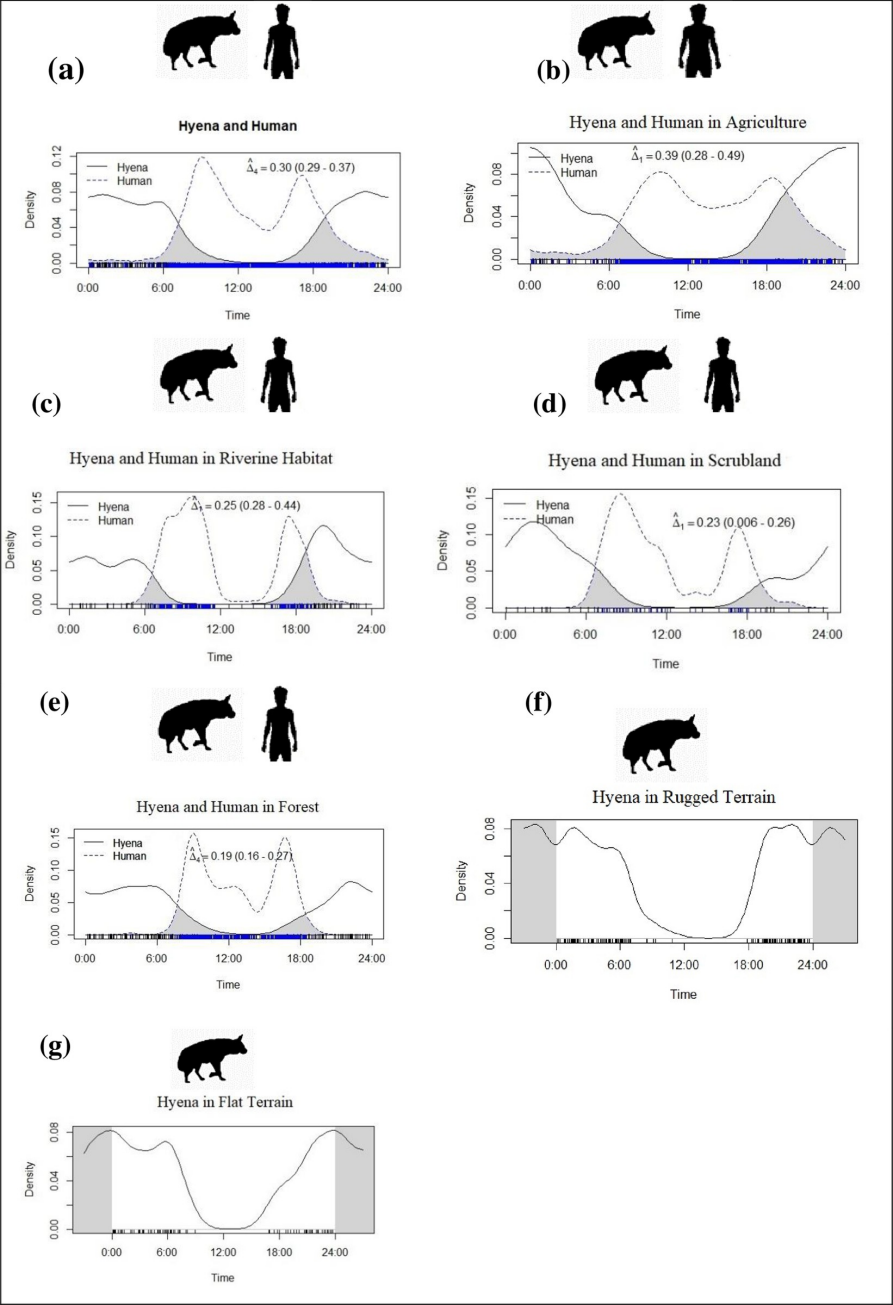
The kernel density function of activity and overlap plot of striped hyena and human in different land-use categories (a) along with human (b) agricultural area (c) riverine area (d) scrubland (e) forest (f) rugged terrain (g) flat terrain, in the human-dominated landscape of Sawai Mansingh Wildlife Sanctuary, Rajasthan.

Striped hyenas were crepuscular and nocturnal, showing bimodal peak activity (Fig 4A). The activity of hyenas was reduced during the daytime when human activities were at their peak (Fig 4A). In all land-use patterns, hyena activity was crepuscular and nocturnal with a bimodal peak of activity. However, there was no sign of activity during the daytime when human activity was high (Fig 4B–4E). The overall activity overlap between humans and hyenas [Δ4 = 0.30, CI = 0.29–0.37, Fig 4A]. The highest activity overlap was observed in the agricultural area [Δ1 = 0.39, CI = 0.28–0.49, Fig 4B], followed by riverine area [Δ1 = 0.25, CI = 0.28–0.44, Fig 4C], scrubland [Δ1 = 0.23, CI = 0.006–0.26, Fig 4D], and forest [Δ4 = 0.19, CI = 0.16–0.27, Fig 4E]. We recorded no major difference in the activity of striped hyenas in both rugged and flat terrain (Fig 4F and 4G). The overall temporal association between human and striped hyenas was calculated at 0.46 (CI = 0.38–0.53, p = <0.05). We observed the highest association between striped hyena and human in agricultural area (0.92 [CI = 0.75–0.99], p = <0.05) followed by riverine area (0.75 [0.63–0.86], p = <0.05), forest (0.66 [0.58–0.73], p = <0.05) and scrubland (0.63 [042–0.82], p = <0.05).

### Spatial use of the landscape by hyena

**Fig 5:**
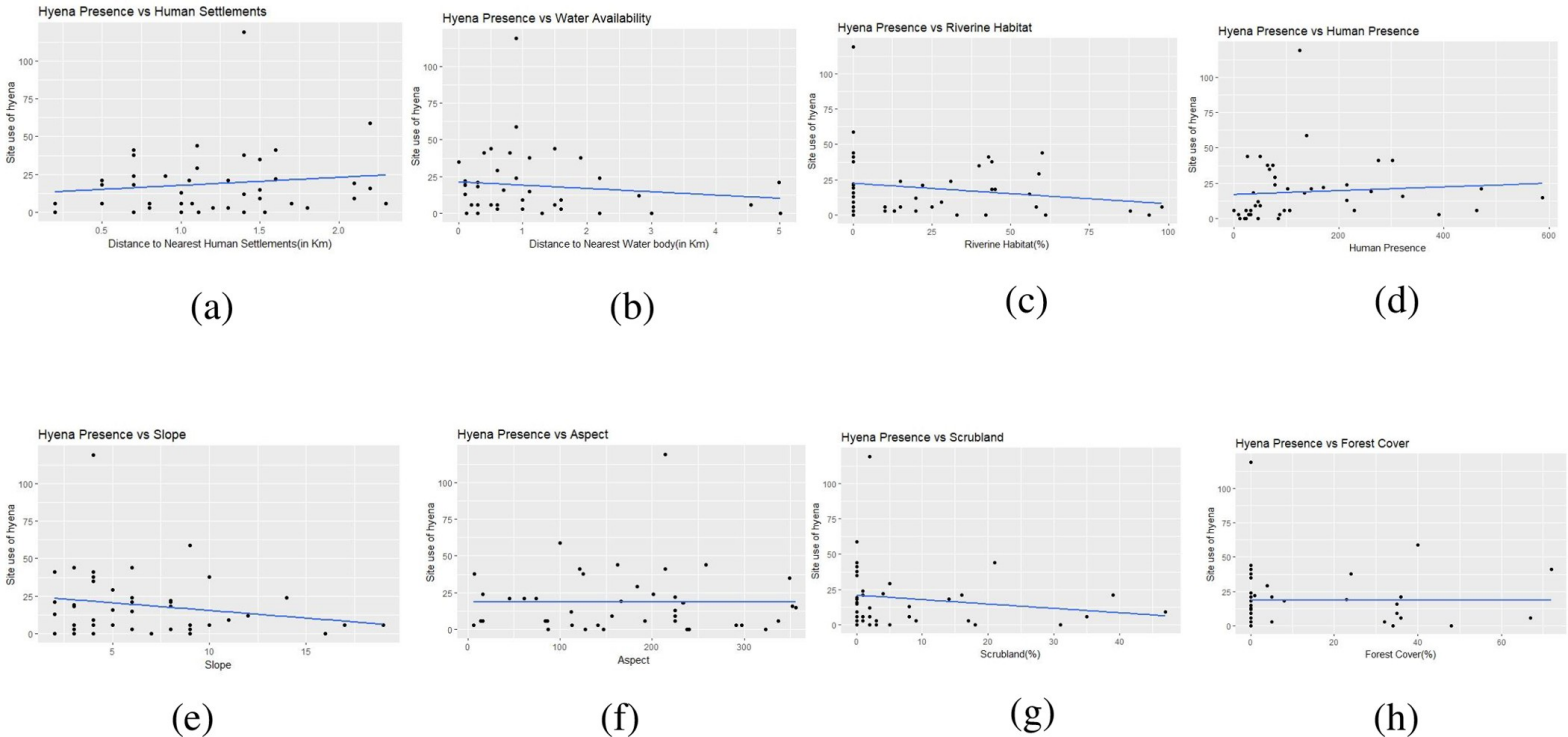
The spatial relationship between the striped hyena and environmental variables (a) hyena vs distance to human settlements or village (km), (b) hyena vs scrubland, (c) hyena vs distance to water (km), (d) hyena vs aspect, (e) hyena vs slope (f) hyena vs riverine area, (g) hyena vs forest cover (h) hyena vs human presence, in the human-dominated landscape of Sawai Mansingh wildlife sanctuary, Rajasthan.

In our analysis, we included all predictor variables: the top three models’ performance with less than an ΔAIC < 2, explained the site used by hyena and selected the top model that had Akaike weights 0.22 (Table 2). The coefficient of predictor variables was generated using model-averaged. The predictor variable, including distance from human settlement, scrubland area, distance from the water, was the best predictor for striped hyena on-site use (Table 3). The distance from the human settlement (β = 0.28, p = <0.05) variable was positively associated with the detection of hyena predicted the detection rate of striped hyena increased with decreasing distance from the human settlement (Fig 5A). While the scrubland (β = -0.01, p = <0.05) and water availability (β = -0.16, p = <0.05) were negatively associated with the detection of hyena (Table 3) (Fig 5B and 5C). The striped hyena capture rate decreased with increasing distance to water (Fig 5C). However, the aspect (β = -0.00, p = >0.05), slope (β = -0.005, p = >0.05), riverine (β = 0.001, p = >0.05) and forest (β = -0.00, p = >0.05) had no significant effect on detection of hyena (Fig 5D–5G). Human presence had no significant effect on detection of hyena (β = -0.00, p = >0.05) (Fig 5H).

**Table 2:** Result of Generalised Linear Model used to evaluate the environmental and anthropogenic variables on-site use by striped hyena in the human-dominated landscape of Sawai Mansingh wildlife sanctuary, Rajasthan. Top candidate models predicting the habitat selection of striped hyaena in the landscape in Sawai Mansingh Wildlife Sanctuary, Rajasthan, India; aspect; area of riverine (arrive); area of scrubland (arscrb); slope; distance from the human settlement (hsdist); distance from water (watdist); area of forest cover (arfcm); human RAI (human).

Covariates	Degree of freedom	log link	AICc	ΔAIC	Weightage of Model
aspect+ arrive + arscrb + hsdist + watdist	6	-478.45	971.23	0.00	0.22
aspect+ arrive + arscrb +hsdist+ slope+ watdist	7	-477.78	972.75	1.53	0.10
aspect+ arscrb +hsdist+ watdist	5	-480.63	972.87	1.65	0.10

**Table 3:** GLM model average coefficient (β) with standard error values (SE) of the variables to explain the site use by striped hyena in the sampling area. * = statistically significant at *P*≤0.05.

Covariates	Coefficient (β)	SE (coefficient)	*P-value*
Intercept	2.894	0.146	0.00***
Aspect	-0.0005	0.00	0.26
Area of riverine habitat	0.0001	0.001	0.28
Area of scrubland	-0.017	0.003	0.00***
Distance from human settlement	0.280	0.073	0.0001***
Distance from the water body	-0.161	0.037	0.00***
Slope	-0.006	0.012	0.62
Forest cover	-0.0001	0.001	0.89
Human presence	-0.00	0.00	0.93

### Local’s perception and livestock density

The people reported no conflict with striped hyenas in and around SMS WLS. Most people (63%) had a positive attitude, while 25.5% had a negative and 11.5% had a neutral attitude toward hyena. A total of 78% of people considered hyena’s role in the ecosystem as cleaning off carrion, while 17.5% people considered predation of livestock and 04% people were not aware of any significant role played by a hyena. In the study area, every month, an average of 40–50 livestock died due to diseases (26%), natural death (36%) or predation by carnivores (13.5%), and carcasses were dumped ~1–2 km distance away from their villages into dumping ground. As per the animal husbandry records, there were 21,272 livestock heads in 22 villages within 75 km^2^. The estimated livestock density in the study area was 283.62 animals/km^2^.

## Discussion

### Striped hyena density

Our results suggest that low-risk conflict species and the scavenging nature of hyena allow them to coexist with humans in the landscape [30]. The density estimates of hyena in our study area were higher than in the adjoining protected area Ranthambhore National Park (5.49 individuals/100 km^2^) [31], but lower than previous studies in similar habitat in Sariska Tiger Reserve (STR) (15.1 individuals/100 km^2^) [59]. However, the hyena densities in the outside protected area in India range from 3.67 to 5.03 individuals/100 km^2^ [30, 60]; whereas estimates for protected areas (i.e., Rajaji National Park and Gir National Park) were 3.91, 6.50 individuals/100 km^2^, respectively in India [61, 62]. Several ecological factors may be driving carnivore densities, with prey abundance among the important factors [63, 64]. STR had the highest wild (107 animals/ km^2^) and domestic (222 animals/km^2^) prey densities [65, 66], which may be the reason for the high density of hyenas. While in Israel density of striped hyenas was higher away from the agriculture and human settlement [67]. In our study area, there are 75 villages located within the five km of boundary of SMS WLS, and the livelihood of locals is primarily dependent on agriculture, and their economy is supplemented by animal husbandry. In our study area, livestock density was estimated to be 283.62 animals/km^2^, and it was observed that 40–50 livestock/month died due to various causes (i.e., starvation, inadequate veterinary care, depredation by tiger or leopard, disease, poor sanitation; personal observation), which were not buried or consumed by locals’ dues to their religious views [68]. The availability of livestock carcasses likely provides subsidised scavenged food sources for striped hyenas in the landscape and lower competition from other predators. Being a specialised scavenger, it was implicit that the striped hyena’s distribution and density might be related to livestock abundance (i.e., carcasses) [30]. However, studies suggested human impact caused low density of striped hyena in East Africa [69]. While in South Africa, the high densities of brown hyenas were observed near cattle farms compared to neighbouring protected areas [70]. Hence the shared landscape provides scavenging opportunities for hyenas, suggesting the higher densities.

### Influence of human activity on striped hyena nocturnality

Temporal avoidance of humans may ease human-hyena coexistence in the shared landscape. Hyenas exhibited nocturnal behaviour in this study. The nocturnal activity of hyenas has been interpreted as a response to human activity, having high human activity during the daytime. We observed that the hyena activity was the crepuscular onset of activity to occur around sunset around 1800 hrs. in comparison, another set of activities occurred around early in the morning at 0600 hrs. In Israel, the striped hyena’s activity was affected by high human activities near agricultural areas [71, 72].

Similarly, our result also revealed the high activity overlapped and high temporal association with humans and hyenas in the agriculture area. In our study area, winter crops (i.e., mustard, chilli, wheat, maze, etc.) were guarded by peoples for protection from wild ungulates (i.e., nilgai and wild boar), and crops provide the cover to hyena for movement in the landscape; hence the overlaps may be expected in agriculture area. While the villages near riverine and scrubland habitats provide abundant food for hyenas, it was common for peoples to discard livestock carcasses and leave a substantive, easily attainable food for hyenas to exploit. People used these habitats for grazing livestock during the daytime, and hyenas utilised areas when people were least likely to be active, i.e., 1800–1900 hrs; hence the activity of hyena was increased during the crepuscular period to benefits of accessing resource subsidies. Likewise, many carnivores (i.e., lion, tiger, wolf, spotted hyenas, brown hyenas) adapt in human-dominated areas to Spatio-temporal avoidance of humans via shifting the activity timing to a preference for nocturnality [9, 25, 73, 74]. The hyena activities were minimally overlapped during 0600 hours to 1200 hours with humans in forest habitat. Due to the forest department restrictions, the human activities are minimal in the forested area; however, the locals illegally graze the cattle and collect firewood for cooking purpose, while hyena uses the forest area for daytime resting and denning purposes [31]. Hyenas are vulnerable to being killed by people and predation by feral dogs. Therefore, they generally avoid human interactions, especially during daylight hours [30, 75].

### Spatial use of the landscape by hyena

Our results are consistent with the general perception that in India, the hyena is recognised as low-risk species compared to other large carnivores and can coexist with humans in a shared landscape [22]. Our results are similar to other studies on habitat selection of hyenas from India [30, 31, 59], Nepal [76], and Africa [20], which found the distance from the human settlement as the significant indicator for hyena detection due to foraging opportunities [30, 31, 77]. Hyenas feed easily on domestic waste in slaughterhouses, garbage dumps, livestock carcasses, and poultry farms [30, 78, 79]. In our study area, due to animal husbandry practices of peoples, generates abundant anthropogenic food (i.e., livestock carcasses). Hyenas likely get most food from scavenging and readily use anthropogenic food in the landscape; hence the detection rate of hyenas is predicted to be high near human settlements. It is often reported that large carnivores (i.e., tiger, leopard, lion) predate on livestock and attack humans, which often leads to conflict [80]. Previous studies reported that the attitude of positive or neutral towards the species is the key factor of coexistence between humans and wildlife [81]. In our study area, people’s attitude towards hyenas was positive, and locals reported no conflict and active livestock predation as they considered the hyena a scavenger. Previous studies reported forest cover and scrubland as important factors for hyena detection [31, 76]. We found that these habitat variables were negatively associated with hyena site use. Scrublands are treated as ‘wastelands’, and people diverted them for commercial use and converted them into agriculture [82]. Hyena prefers open or thorn habitats in arid and semiarid environments [32, 76, 83], and the presence of the hyena may have been associated with such habitats because hyenas used scrubland for movement. Habitat use studies indicate the distance to the water body and riverine habitat found important factors for site use by hyenas [76]. Our results generally agree with this pattern in the area both humans and hyenas temporally used the same water resources. The rugged and gullied terrain of riverine habitat provides a suitable denning site to hyenas and easy access to the water body from the nearby Chambal river. The riverine habitats are barren areas which are alluvium deposited by the Chambal river itself [84], have less interest to villagers but are used by different faunal species for denning. A previous study suggested the rugged terrain in the landscape provides disturbance-free denning refugia (i.e., not used by livestock, humans, or guard dogs) of hyenas may provide optimal conditions for breeding and raising pups [30, 31]. Our study demonstrates that the scrubland, agricultural lands, and riverine habitats may serve as supplementary habitats for hyenas. The coexistence of hyenas and humans in the shared landscape is supported by mutual benefits, where hyenas get benefits from food and humans’ benefit from waste removal. Thus, sharing landscape in a human-dominated landscape without negatively impacting each other is a possible key factor of human-wildlife coexistence [81].

## Supporting information

S1 Appendix Data analysis codes of R used in the paper. (DOCX)

S2 Appendix Data used for analysis. (XLSX)

S1 Text (DOCX)

## References

[1] Barnosky AD, Hadly EA, Bascompte J, Berlow EL, Brown JH, Fortelius M, et al. Approaching a state shift in Earth’s biosphere. Nature. 2012 Jun;486(7401):52–8. doi: 10.1038/nature11018 22678279

[2] Ellis EC, Fuller DQ, Kaplan JO, Lutters WG, Blum JD. Dating the Anthropocene: Towards an empirical global history of human transformation of the terrestrial biosphere Dating the Anthropocene. Elementa: Science of the Anthropocene. 2013 Jan 1;1.

[3] Tucker MA, Böhning-Gaese K, Fagan WF, Fryxell JM, Van Moorter B, Alberts SC, et al. Moving in the Anthropocene: Global reductions in terrestrial mammalian movements. Science. 2018 Jan 26;359(6374):466–9. doi: 10.1126/science.aam9712 29371471

[4] Galán-Acedo C, Arroyo-Rodríguez V, Andresen E, Arregoitia LV, Vega E, Peres CA, et al. The conservation value of human-modified landscapes for the world’s primates. Nature communications. 2019 Jan 11;10(1):1–8. doi: 10.1038/s41467-018-07882-8 30635587 PMC6329842

[5] Lamb CT, Ford AT, McLellan BN, Proctor MF, Mowat G, Ciarniello L, et al. The ecology of human-carnivore coexistence. Proceedings of the National Academy of Sciences. 2020 Jul 28;117(30):17876–83. doi: 10.1073/pnas.1922097117 32632004 PMC7395549

[6] Carter NH, Linnell JD. Co-adaptation is key to coexisting with large carnivores. Trends in Ecology & Evolution. 2016 Aug 1;31(8):575–8. doi: 10.1016/j.tree.2016.05.006 27377600

[7] Ripple WJ, Estes JA, Beschta RL, Wilmers CC, Ritchie EG, Hebblewhite M, et al. Status and ecological effects of the world’s largest carnivores. Science. 2014 Jan 10;343(6167). doi: 10.1126/science.1241484 24408439

[8] Treves A, Bruskotter J. Tolerance for predatory wildlife. Science. 2014 May 2;344(6183):476–7. doi: 10.1126/science.1252690 24786065

[9] Carter NH, Shrestha BK, Karki JB, Pradhan NM, Liu J. Coexistence between wildlife and humans at fine spatial scales. Proceedings of the National Academy of Sciences. 2012 Sep 18;109(38):15360–5. doi: 10.1073/pnas.1210490109 22949642 PMC3458348

[10] Gilbert SL, Sivy KJ, Pozzanghera CB, DuBour A, Overduijn K, Smith MM, et al. Socioeconomic benefits of large carnivore recolonisation through reduced wildlife‐vehicle collisions. Conservation Letters. 2017 Jul;10(4):431–9.

[11] Lozano J, Olszańska A, Morales-Reyes Z, Castro AA, Malo AF, Moleón M, et al. Human-carnivore relations: a systematic review. Biological Conservation. 2019 Sep 1;237:480–92.

[12] Decker DJ, Chase LC. Human dimensions of living with wildlife: a management challenge for the 21st century. Wildlife Society Bulletin. 1997 Dec 1:788–95.

[13] Nyhus PJ. Human-wildlife conflict and coexistence. Annual review of environment and resources. 2016 Oct 17;41:143–71.

[14] Morehouse AT, Boyce MS. Troublemaking carnivores: conflicts with humans in a diverse assemblage of large carnivores. Ecology and Society. 2017 Sep 1;22(3).

[15] Chapron G, Kaczensky P, Linnell JD, Von Arx M, Huber D, Andrén H, et al. Recovery of large carnivores in Europe’s modern human-dominated landscapes. science. 2014 Dec 19;346(6216):1517–9. doi: 10.1126/science.1257553 25525247

[16] Dorling D. World population prospects at the UN: our numbers are not our problem?. The Struggle for Social Sustainability: Moral Conflicts in Global Social Policy. 2021 Apr 28:129.

[17] Watling JI, Nowakowski AJ, Donnelly MA, Orrock JL. Meta‐analysis reveals the importance of matrix composition for animals in fragmented habitat. Global Ecology and Biogeography. 2011 Mar;20(2):209–17.

[18] Corlett RT. The Anthropocene concept in ecology and conservation. Trends in ecology & evolution. 2015 Jan 1;30(1):36–41. doi: 10.1016/j.tree.2014.10.007 25468360

[19] O’Bryan CJ, Braczkowski AR, Beyer HL, Carter NH, Watson JE, McDonald-Madden E. The contribution of predators and scavengers to human well-being. Nature Ecology & evolution. 2018 Feb;2(2):229–36. 29348647 10.1038/s41559-017-0421-2

[20] Yirga G, Leirs H, De Iongh HH, Asmelash T, Gebrehiwot K, Deckers J, et al. Spotted hyena (Crocuta crocuta) concentrate around urban waste dumps across Tigray, northern Ethiopia. Wildlife Research. 2015 Jul 17;42(7):563–9.

[21] Li J, Wang D, Yin H, Zhaxi D, Jiang Z, Schaller GB, et al. Role of Tibetan Buddhist monasteries in snow leopard conservation. Conservation biology. 2014 Feb;28(1):87–94. doi: 10.1111/cobi.12135 23992599

[22] Srivathsa A, Puri M, Karanth KK, Patel I, Kumar NS. Examining human-carnivore interactions using a socio-ecological framework: sympatric wild canids in India as a case study. Royal Society open science. 2019 May 29;6(5):182008. doi: 10.1098/rsos.182008 31218031 PMC6549949

[23] Ghosal S, Athreya VR, Linnell JD, Vedeld PO. An ontological crisis? A review of large felid conservation in India. Biodiversity and Conservation. 2013 Oct;22(11):2665–81.

[24] Singh R, Pandey P, Qureshi Q, Sankar K, Krausman PR, Goyal SP. Philopatric and natal dispersal of tigers in a semiarid habitat, western India. Journal of Arid Environments. 2021 Jan 1;184:104320.

[25] Gaynor KM, Hojnowski CE, Carter NH, Brashares JS. The influence of human disturbance on wildlife nocturnality. Science. 2018 Jun 15;360(6394):1232–5. doi: 10.1126/science.aar7121 29903973

[26] Gehrt SD. Urban carnivores. Johns Hopkins university press; 2010.

[27] Redpath SM, Young J, Evely A, Adams WM, Sutherland WJ, Whitehouse A, et al. Understanding and managing conservation conflicts. Trends in ecology & evolution. 2013 Feb 1;28(2):100–9. doi: 10.1016/j.tree.2012.08.021 23040462

[28] AbiSaid M, Dloniak SM. Hyaena hyaena. The IUCN Red List of Threatened Species. 2015;444:2015.

[29] Califf KJ, Green DS, Wagner AP, Scribner KT, Beatty K, Wagner ME, et al. Genetic relatedness and space use in two populations of striped hyenas (Hyaena hyaena). Journal of Mammalogy. 2020 Apr 8;101(2):361–72.

[30] Singh P, Gopalaswamy AM, Karanth KU. Factors influencing densities of striped hyenas (Hyaena hyaena) in arid regions of India. Journal of Mammalogy. 2010 Oct 15;91(5):1152–9.

[31] Singh R, Qureshi Q, Sankar K, Krausman PR, Goyal SP, Nicholson KL. Population density of striped hyenas in relation to habitat in a semiarid landscape, western India. Acta Theologica. 2014 Oct 1;59(4):521–7.

[32] Kruuk H. Feeding and social behaviour of the striped hyaena (Hyaena Vulgaris Desmarest). African Journal of Ecology. 1976 Jun;14(2):91–111.

[33] Singh R, Qureshi Q, Sankar K, Krausman PR, Goyal SP. Use of camera traps to determine dispersal of tigers in semiarid landscape, western India. Journal of arid environments. 2013 Nov 1;98:105–8.

[34] Jhala YV, Qureshi Q, Gopal R, Sinha PR (Eds.). Status of tigers, co-predators and prey in India, 2010 National Tiger Conservation Authority, Government of India, New Delhi. 2011.

[35] Singh R, Nigam P, Goyal SP, Joshi BD, Sharma S, Shekhawat RS. Survival of dispersed orphaned cubs of tiger (Panthera tigris Tigris) in fragmented habitat of Ranthambhore Tiger Reserve In India. Indian Forester. 2011 Oct 1;137(10):1171–6.

[36] Rodgers WA, Panwar HS. Planning a wildlife protected area network in India. 1988.

[37] Rajasthan Population Census, India Village wise-Population 2011 http://rdprd.gov.in/ Accessed Aug 16 2021.

[38] Champion HG, Seth SK. A revised survey of the forest types of India. Manager of publications; 1968.

[39] Efford MG. Estimation of population density by spatially explicit capture-recapture analysis of data from area searches. Ecology. 2011 Dec;92(12):2202–7. doi: 10.1890/11-0332.1 22352159

[40] Efford MG, Dawson DK, Robbins CS. DENSITY: software for analysing capture-recapture data from passive detector arrays. Animal Biodiversity and Conservation. 2004;27(1):217–28.

[41] Efford M. A tutorial on fitting spatially explicit capture-recapture models in secr,2019b.

[42] Efford M. Density estimation in live‐trapping studies. Oikos. 2004 Sep;106(3):598–610, 2004.

[43] Zimmermann F, Breitenmoser‐Würsten C, Molinari‐Jobin A, Breitenmoser U. Optimising the size of the area surveyed for monitoring a Eurasian lynx (Lynx lynx) population in the Swiss Alps by means of photographic capture-recapture. Integrative Zoology. 2013 Sep;8(3):232–43. doi: 10.1111/1749-4877.12017 24020463

[44] Farris ZJ, Gerber BD, Karpanty S, Murphy A, Andrianjakarivelo V, Ratelolahy F, et al. When carnivores roam: temporal patterns and overlap among M Madagascar’s native and exotic carnivores. Journal of Zoology. 2015 May;296(1):45–57.

[45] Chaudhary R, Zehra N, Musavi A, Khan JA. Spatio-temporal partitioning and coexistence between leopard (Panthera pardus fusca) and Asiatic lion (Panthera leo persica) in Gir protected area, Gujarat, India. PloS one. 2020 Mar 11;15(3):e0229045. doi: 10.1371/journal.pone.0229045 32160193 PMC7065753

[46] Cramér H. A contribution to the theory of statistical estimation. Scandinavian Actuarial Journal. 1946 Jan 1;1946(1):85–94.

[47] Ben-Shachar MS, Lüdecke D, Makowski D. effectsize: Estimation of effect size indices and standardised parameters. Journal of Open Source Software. 2020 Dec 23;5(56):2815.

[48] Linkie M, Ridout MS. Assessing tiger–prey interactions in Sumatran rainforests. Journal of Zoology. 2011 Jul;284(3):224–9.

[49] Meredith M, Ridout M. Overview of the overlap package. R. Proj. 2021

[50] Numbisi FN, Alemagi D, Degrande A, Van Coillie F. Farm rejuvenation-induced changes in tree spatial pattern and live biomass species of Cocoa Agroforests in Central Cameroon: insights for tree conservation incentives in cocoa landscapes. Sustainability 2021, 13, 8483.

[51] Warton DI, Lyons M, Stoklosa J, Ives AR. Three points to consider when choosing a LM or GLM test for count data. Methods in Ecology and Evolution. 2016 Aug;7(8):882–90.

[52] Palmer MS, Swanson A, Kosmala M, Arnold T, Packer C. Evaluating relative abundance indices for terrestrial herbivores from large‐scale camera trap surveys. African journal of ecology. 2018 Dec;56(4):791–803.

[53] Zuur AF, Ieno EN, Elphick CS. A protocol for data exploration to avoid common statistical problems. Methods in ecology and evolution. 2010 Mar;1(1):3–14.

[54] Guisan A, Edwards TC Jr, Hastie T. Generalized linear and generalised additive models in studies of species distributions: setting the scene. Ecological modelling. 2002 Nov 30;157(2–3):89–100.

[55] Guerisoli MD, Caruso N, Luengos Vidal EM, Lucherini M. Habitat use and activity patterns of Puma concolor in a human-dominated landscape of central Argentina. Journal of Mammalogy. 2019 Feb 28;100(1):202–11.

[56] Burnham KP, Anderson DR. A practical information-theoretic approach. Model selection and multimodel inference. 2002;2.

[57] Symonds MR, Moussalli A. A brief guide to model selection, multimodel inference and model averaging in behavioural ecology using Akaike’s information criterion. Behavioral Ecology and Sociobiology. 2011 Jan;65(1):13–21.

[58] Welch RJ, Tambling CJ, Bissett C, Gaylard A, Müller K, Slater K, et al. Brown hyena habitat selection varies among sites in a semiarid region of southern Africa. Journal of Mammalogy. 2016 Mar 23;97(2):473–82.

[59] Gupta S, Mondal K, Sankar K, Qureshi Q. Estimation of striped hyena Hyaena hyaena population using camera traps in Sariska Tiger Reserve, Rajasthan, India. Journal of the Bombay Natural History Society. 2009 Sep;106(3):284.

[60] Athreya V, Odden M, Linnell JD, Krishnaswamy J, Karanth U. Big cats in our backyards: persistence of large carnivores in a human-dominated landscape in India. PloS one. 2013 Mar 6;8(3):e57872. doi: 10.1371/journal.pone.0057872 23483933 PMC3590292

[61] Harihar A, Ghosh M, Fernandes M, Pandav B, Goyal SP. Use of photographic capture-recapture sampling to estimate density of Striped Hyena (Hyaena hyaena): implications for conservation, 2010.

[62] Alam S. *Status, Ecology and conservation of striped Hyena Hyaena hyaena in Gir National park and Sanctuary, Gujrat* (Doctoral dissertation, Aligarh Muslim University), 2011.

[63] Hayward MW, O’Brien J, Kerley GI. Carrying capacity of large African predators: predictions and tests. Biological Conservation. 2007 Sep 1;139(1–2):219–29.

[64] Faure JP, Swanepoel LH, Cilliers D, Venter JA, Hill RA. Estimates of carnivore densities in a human-dominated agricultural matrix in South Africa. Oryx. 2021:1–8.

[65] Sankar K, Qureshi Q, Nigam P, Malik PK, Sinha PR, Mehrotra RN, et al. Monitoring of reintroduced tigers in Sariska Tiger Reserve, Western India: preliminary findings on home range, prey selection and food habits. Tropical Conservation Science. 2010 Sep;3(3):301–18.

[66] Avinandan D, Sankar K, Qureshi QA. Prey selection by tigers (Pantheratigristigris) in Sariska tiger reserve, Rajasthan, India. Journal of the Bombay Natural History Society. 2008 Sep;105(3):247–54.

[67] Shamoon H, Shapira I. Limiting factors of Striped Hyaena, Hyaena hyaena, distribution and densities across climatic and geographical gradients (Mammalia: Carnivora). Zoology in the Middle East. 2019 Jul 3;65(3):189–200.

[68] Chhangani AK. Present status of vultures in the Great Indian Thar Desert. In Faunal ecology and conservation of the Great Indian Desert 2009 (pp. 65–83). Springer, Berlin, Heidelberg.

[69] Hardouin M, Searle CE, Strampelli P, Smit J, Dickman A, Lobora AL, et al. Density responses of lesser-studied carnivores to habitat and management strategies in southern Tanzania’s Ruaha-Rungwa landscape. PloS one. 2021 Mar 30;16(3):e0242293. doi: 10.1371/journal.pone.0242293 33784297 PMC8009394

[70] Kent VT, Hill RA. The importance of farmland for the conservation of the brown hyaena Parahyaena brunnea. Oryx. 2013 Jul;47(3):431–40.

[71] Rosenberg B, Reichman A, Shamoon H. Striped hyena (Hyaena hyaena) movement patterns near Haifa city, Mt. Carmel, Israel. Jerusalem. 2016.

[72] Shamoon H, Cain S, Shanas U, Bar-Massada A, Malihi Y, Shapira I. Spatio-temporal activity patterns of mammals in an agro-ecological mosaic with seasonal recreation activities. European Journal of Wildlife Research. 2018 Jun;64(3):1–0.

[73] Ashenafi ZT, Coulson T, Sillero-Zubiri C, Leader-Williams N. Behaviour and ecology of the Ethiopian wolf (Canis simensis) in a human-dominated landscape outside protected areas. InAnimal Conservation forum 2005 May (Vol. 8, No. 2, pp. 113–121). Cambridge University Press.

[74] Hebblewhite M, Merrill E. Modelling wildlife-human relationships for social species with mixed‐effects resource selection models. Journal of applied ecology. 2008 Jun;45(3):834–44.

[75] Mills G, Hofer H. Hyaenas: status survey and conservation action plan. IUCN, Gland (Suiza). SSC Hyaena Specialist Group; 1998.

[76] Bhandari S, Bhusal DR, Psaralexi M, Sgardelis S. Habitat preference indicators for striped hyena (Hyaena hyaena) in Nepal. Global Ecology and Conservation. 2021 Jun 1;27:e01619.

[77] Akay AE, Inac S, Yildirim IC. Monitoring the local distribution of striped hyenas (Hyaena hyaena L.) in the Eastern Mediterranean Region of Turkey (Hatay) by using GIS and remote sensing technologies. Environmental monitoring and assessment. 2011 Oct;181(1):445–55. doi: 10.1007/s10661-010-1840-6 21181258

[78] Abi-Said MR, Abi-Said DM. Distribution of the Striped Hyaena (Hyaena hyaena syriaca Matius, 1882)(Carnivora: Hyaenidae) in urban and rural areas of Lebanon. Zoology in the Middle East. 2007 Jan 1;42(1):3–14.

[79] Alam MS, Khan JA, Kushwaha SP, Agrawal R, Pathak BJ, Kumar S. Assessment of suitable habitat of near threatened Striped Hyena (Hyaena hyaena Linnaeus, 1758) using remote sensing and geographic information system. Asian Journal of Geoinformatics. 2014 Jul 16;14(2).

[80] Singh R, Nigam P, Qureshi Q, Sankar K, Krausman PR, Goyal SP, et al. Characterising human-tiger conflict in and around Ranthambhore Tiger Reserve, western India. European Journal of Wildlife Research. 2015 Apr;61(2):255–61.

[81] Glikman JA, Frank B, Ruppert KA, Knox J, Sponarski CC, Metcalf EC, et al. Coexisting with different human-wildlife coexistence perspectives. Frontiers in Conservation Science. 2021:75.

[82] Singh P, Rahmani AR, Wangchuk S, Mishra C, Singh KD, Narain P, et al. Report of the task force on grasslands and deserts. Planning Commission, Government of India, New Delhi. 2006.

[83] Leakey LN, Milledge SA, Leakey SM, Edung J, Haynes P, Kiptoo DK, et al. Diet of striped hyaena in northern Kenya. African Journal of Ecology. 1999 Sep;37(3):314–26.

[84] Ranga V, Van Rompaey A, Poesen J, Mohapatra SN, Pani P. Semi-automatic delineation of badlands using contrast in vegetation activity: a case study in the lower Chambal valley, India. Geocarto International. 2015 Sep 14;30(8):919–36.

