# Supplementary material for: High striped hyena density suggests coexistence with humans in an agricultural landscape, Rajasthan
Source: PLoS One. 2022 May 4;17(5):e0266832. doi: 10.1371/journal.pone.0266832 (PMC9067646; doi:10.1371/journal.pone.0266832)
Supplement: S1 Appendix — (DOCX) [file pone.0266832.s001.docx]

**Appendix A1**: Analysis R Codes for Activity Overlap of Hyena and Human in different land use categories, GLM

#Activity Overlap Code

library(overlap)

setwd("D:\\Debashish Laptop Data\\Camera Trap Activity-Landuse Type")

dir()

Hyena<-read.csv("hyena.CSV")

Hyena<-Hyena$Time*2*pi

densityPlot(Hyena)

densityPlot(Hyena, linet = c(1), linec = c("red"), linewidth = c (2,2), rug = TRUE)

Hyena_Forest<-read.csv("foresth.CSV")

Hyena_Forest<-Hyena_Forest$Time*2*pi

densityPlot(Hyena_Forest)

densityPlot(Hyena_Forest, linet = c(1), linec = c("red"), linewidth = c (2,2), rug = TRUE)

Hyena_Agriculture<-read.csv("agricultureh.CSV")

Hyena_Agriculture<-Hyena_Agriculture$Time*2*pi

densityPlot(Hyena_Agriculture)

densityPlot(Hyena_Agriculture, linet = c(1), linec = c("red"), linewidth = c (2,2), rug = TRUE)

Hyena_Riverine<-read.csv("riverineh.CSV")

Hyena_Riverine<-Hyena_Riverine$Time*2*pi

densityPlot(Hyena_Riverine

densityPlot(Hyena_Riverine, linet = c(1), linec = c("red"), linewidth = c (2,2), rug = TRUE)

Hyena_Scrubland<-read.csv("scrublandh.CSV")

Hyena_Scrubland<-Hyena_Scrubland$Time*2*pi

densityPlot(Hyena_Scrubland)

densityPlot(Hyena_Scrubland, linet = c(1), linec = c("red"), linewidth = c (2,2), rug = TRUE)

#Human

Human<-read.csv("Human.CSV")

Human<-Human$Time*2*pi

densityPlot(Human)

densityPlot(Human, linet = c(1), linec = c("red"), linewidth = c (2,2), rug = TRUE)

Human_Forest<-read.csv("Human_F.CSV")

Human_Forest<-Human_Forest$Time*2*pi

densityPlot(Human_Forest)

densityPlot(Human_Forest, linet = c(1), linec = c("red"), linewidth = c (2,2), rug = TRUE)

Human_Agriculture<-read.csv("Human_A.CSV")

Human_Agriculture<-Human_Agriculture$Time*2*pi

densityPlot(Human_Agriculture)

densityPlot(Human_Agriculture, linet = c(1), linec = c("red"), linewidth = c (2,2), rug = TRUE)

Human_Riverine<-read.csv("Human_R.CSV")

Human_Riverine<-Human_Riverine$Time*2*pi

densityPlot(Human_Riverine)

densityPlot(Human_Riverine, linet = c(1), linec = c("red"), linewidth = c (2,2), rug = TRUE)

Human_Scrubland<-read.csv("Human_S.CSV")

Human_Scrubland<-Human_Scrubland$Time*2*pi

densityPlot(Human_Scrubland)

densityPlot(Human_Scrubland, linet = c(1), linec = c("red"), linewidth = c (2,2), rug = TRUE)

#overlapforest

overlapPlot(Hyena_Forest,Human_Forest)

overlapPlot(Hyena_Forest, Human_Forest, xcenter = "n", linet = c(1,2), linec = c("black", "blue"), linewidth = c(1,1), rug=TRUE)

min(length(Hyena_Forest), length(Human_Forest))

APest <- overlapEst(Hyena_Forest, Human_Forest, type = "Dhat4")

APest

Hyena_Forest <- resample(Hyena_Forest, 10000)

dim(Hyena_Forest)

Human_Forest <-resample(Human_Forest, 10000)

dim(Human_Forest)

HFHF <- bootEst(Hyena_Forest, Human_Forest, adjust = c(0.8, 1, 4))

dim(HFHF)

BSmean <- colMeans(HFHF)

BSmean

tmp <- HFHF[,1]

bootCI(HFHF[1], tmp)

bootEst(Hyena_Forest, Human_Forest, type=("Dhat4"))

sd(bootEst(Hyena_Forest, Human_Forest, type=("Dhat4")))

legend("topright", c("Hyena_Forest", "Human_Forest"), lty=c(1,2), col=c("black", "blue"), bg="white", bty='n', y.intersp=1)

text(4,0.135,substitute(paste(hat(Delta))[4]=='0.17 (0.11 - 0.21)'))

#overlap agriculture

overlapPlot(Hyena_Agriculture,Human_Agriculture)

overlapPlot(Hyena_Agriculture, Human_Agriculture, xcenter = "n", linet = c(1,2), linec = c("black", "blue"), linewidth = c(1,1), rug=TRUE)

min(length(Hyena_Agriculture), length(Human_Agriculture))

APest <- overlapEst(Hyena_Agriculture, Human_Agriculture, type = "Dhat1")

APest

Hyena_Agriculture <- resample(Hyena_Agriculture, 10000)

dim(Hyena_Agriculture)

Human_Agriculture <-resample(Human_Agriculture, 10000)

dim(Human_Agriculture)

HAHA <- bootEst(Hyena_Agriculture, Human_Agriculture, adjust = c(0.8, 1, 4))

dim(HAHA)

BSmean <- colMeans(HAHA)

BSmean

tmp <- HAHA[,1]

bootCI(HAHA[1], tmp)

bootEst(Hyena_Agriculture, Human_Agriculture, type=("Dhat1"))

sd(bootEst(Hyena_Agriculture, Human_Agriculture, type=("Dhat1")))

legend("topleft", c("Hyena", "Human"), lty=c(1,2), col=c("black", "blue"), bg="white", bty='n', y.intersp=1)

text(12,0.10,substitute(paste(hat(Delta))[1]=='0.39 (0.28 - 0.49)'))

#overlap riverine

overlapPlot(Hyena_Riverine,Human_Riverine)

overlapPlot(Hyena_Riverine, Human_Riverine, xcenter = "n", linet = c(1,2), linec = c("black", "blue"), linewidth = c(1,1), rug=TRUE)

min(length(Hyena_Riverine), length(Human_Riverine))

APest <- overlapEst(Hyena_Riverine, Human_Riverine, type = "Dhat1")

APest

Hyena_Riverine <- resample(Hyena_Riverine, 10000)

dim(Hyena_Riverine)

Human_Riverine <-resample(Human_Riverine, 10000)

dim(Human_Riverine)

HRHR <- bootEst(Hyena_Riverine, Human_Riverine, adjust = c(0.8, 1, 4))

dim(HRHR)

BSmean <- colMeans(HRHR)

BSmean

tmp <- HRHR[,1]

bootCI(HRHR[1], tmp)

bootEst(Hyena_Riverine, Human_Riverine, type=("Dhat1"))

sd(bootEst(Hyena_Riverine, Human_Riverine, type=("Dhat1")))

legend("topleft", c("Hyena", "Human"), lty=c(1,2), col=c("black", "blue"), bg="white", bty='n', y.intersp=1)

text(14,0.15,substitute(paste(hat(Delta))[1]=='0.25 (0.28 - 0.44)'))

#overlap scrubland

overlapPlot(Hyena_Scrubland,Human_Scrubland)

overlapPlot(Hyena_Scrubland, Human_Scrubland, xcenter = "n", linet = c(1,2), linec = c("black", "blue"), linewidth = c(1,1), rug=TRUE)

min(length(Hyena_Scrubland), length(Human_Scrubland))

APest <- overlapEst(Hyena_Scrubland, Human_Scrubland, type = "Dhat1")

APest

Hyena_Scrubland <- resample(Hyena_Scrubland, 10000)

dim(Hyena_Scrubland)

Human_Scrubland <-resample(Human_Scrubland, 10000)

dim(Human_Scrubland)

HSHS <- bootEst(Hyena_Scrubland, Human_Scrubland, adjust = c(0.8, 1, 4))

dim(HSHS)

BSmean <- colMeans(HSHS)

BSmean

tmp <- HSHS[,1]

bootCI(HSHS[1], tmp)

bootEst(Hyena_Scrubland, Human_Scrubland, type=("Dhat1"))

sd(bootEst(Hyena_Scrubland, Human_Scrubland, type=("Dhat1")))

legend("topleft", c("Hyena", "Human"), lty=c(1,2), col=c("black", "blue"), bg="white", bty='n', y.intersp=1)

text(17,0.135,substitute(paste(hat(Delta))[1]=='0.21 (0.02 - 0.27)'))

#overlap human and hyena

overlapPlot(Hyena,Human)

overlapPlot(Hyena, Human, xcenter = "n", linet = c(1,2), linec = c("black", "blue"), linewidth = c(1,1), rug=TRUE)

min(length(Hyena), length(Human))

APest <- overlapEst(Hyena, Human, type = "Dhat4")

APest

Hyena <- resample(Hyena, 10000)

dim(Hyena)

Human <-resample(Human, 10000)

dim(Human)

HH <- bootEst(Hyena, Human, adjust = c(0.8, 1, 4))

dim(HH)

BSmean <- colMeans(HH)

BSmean

tmp <- HH[,1]

bootCI(HH[1], tmp)

bootEst(Hyena, Human, type=("Dhat4"))

sd(bootEst(Hyena, Human, type=("Dhat4")))

legend("topright", c("Hyena", "Human"), lty=c(1,2), col=c("black", "blue"), bg="white", bty='n', y.intersp=1)

text(3,0.10,substitute(paste(hat(Delta))[4]=='0.29 (0.28 - 0.37)'))

**#GLM**

env_variables <-read.csv("env_var1.csv")

library(MuMIn)

data.frame(env_variables)

model1 <-glm(hyena ~ water + village + slope + aspect + scrub + forest + riverine + human, data = env_variables, family = poisson(link = 'log'))

summary(model1)

options(na.action = "na.fail")

results<-dredge(model1)

results

mod.avg.hyena<-model.avg(results, fit = TRUE, subset = cumsum(weight) <= .90)

summary(mod.avg.hyena)

Scatter Plot#

library(ggplot2)

ggplot(data = env_variables,mapping = aes(x = forest, y = hyena)) + geom_point() +

labs(x = 'Forest', y = 'Site use of hyena', title = 'Hyena Presence vs Forest') +

geom_smooth(method = 'lm', se = FALSE)

ggplot(data = env_variables,mapping = aes(x = scrub, y = hyena)) + geom_point() +

labs(x = 'Scrubland', y = 'Site use of hyena', title = 'Hyena Presence vs Scrubland') +

geom_smooth(method = 'lm', se = FALSE)

ggplot(data = env_variables,mapping = aes(x = riverine, y = hyena)) + geom_point() +

labs(x = 'Riverine', y = 'Site use of hyena', title = 'Hyena Presence vs Riverine Habitat') +

geom_smooth(method = 'lm', se = FALSE)

ggplot(data = env_variables,mapping = aes(x = water, y = hyena)) + geom_point() +

labs(x = 'Water', y = 'Site use of hyena', title = 'Hyena Presence vs Water Availability') +

geom_smooth(method = 'lm', se = FALSE)

ggplot(data = env_variables,mapping = aes(x = village, y = hyena)) + geom_point() +

labs(x = 'Village', y = 'Site use of hyena', title = 'Hyena Presence vs Human Settlements') +

geom_smooth(method = 'lm', se = FALSE)

ggplot(data = env_variables,mapping = aes(x = slope, y = hyena)) + geom_point() +

labs(x = 'Slope', y = 'Site use of hyena', title = 'Hyena Presence vs Slope') +

geom_smooth(method = 'lm', se = FALSE)

ggplot(data = env_variables,mapping = aes(x = aspect, y = hyena)) + geom_point() +

labs(x = 'Aspect', y = 'Site use of hyena', title = 'Hyena Presence vs Aspect') +

geom_smooth(method = 'lm', se = FALSE)

ggplot(data = env_variables,mapping = aes(x = human, y = hyena)) + geom_point() +

labs(x = 'Human', y = 'Site use of hyena', title = 'Hyena Presence vs Human Presence') +

geom_smooth(method = 'lm', se = FALSE)
