# Supplementary material for: High striped hyena density suggests coexistence with humans in an agricultural landscape, Rajasthan
Source: PLoS One. 2022 May 4;17(5):e0266832. doi: 10.1371/journal.pone.0266832 (PMC9067646; doi:10.1371/journal.pone.0266832)
Supplement: S1 Text — (DOCX) [file pone.0266832.s003.docx]

**High Striped Hyena Density Suggests Co-existence with Humans in an Agricultural Landscape, Rajasthan**

**Author(s):** Debashish Panda^1^, Subham Mohanty^2^, Tanuj Suryan^1^, Puneet Pandey^1,3^*, Hang Lee^3^, Randeep Singh^1*^

**Supporting information:**

**S1 File: Methods**

**Landscape characterization**

To map the potential hyena habitat and land cover types of the landscape, we used an optical remote sensing sentinel-2B multispectral satellite data. We used 2 (blue), 3 (green), 4 (red), and 8 (near-infrared) bands having high spatial resolution (10 m), as it is easily used to identify natural habitats from human-modified habitats [1]. Sentinel-2B data was downloaded on 27 December 2020 from the open-source portal of the US Geological Survey (https://earthexplorer.usgs.gov). We used maximum likelihood classification in the supervised classification method using ArcMap v.10.2.2 [2]. Five major land cover types, i.e., water, forest, agriculture, riverine, and scrubland, were identified (S1 Table). Image elements like tone, texture, shape, size, shadow, location, and association were used for delineated this purpose. Finally, the classification accuracy was evaluated for classification accuracy using a second set of field data.

**Environmental Variables**

**Distance from human settlements**

The location of each village in the landscape was identified by satellite image, google earth, and toposheet (1:50 000 scales) produced by the Survey of India, Govt. of India. Hyenas generally avoid human settlements, but they were attracted as availability of livestock carcasses near settlements as a food subsidiary [3]. Hence, we considered the distance to the nearest human settlement as an important variable. The settlement buffer zone was created and Euclidean distance for each hyena location was extracted using Arc GIS spatial analyst tools.

**Distance to water and river**

In the landscape, drainage lines, water bodies, and the river often provide essential water, movement paths, and foraging for the hyena. The study area is part of the ravine landscape of river Chambal, the gullied terrain near to river Chambal, and the banks of its tributaries providing the refugia site to hyenas. We calculated the Euclidean distance to drainage lines (m), water body (m), and river for each camera trap site using Arc GIS spatial analyst tools.

**Slope and Aspect**

The rugged terrain (slope) provides optimal refuges and denning sites to striped hyenas [4]. The moderate degree of slope is assumed to be more suitable for large mammals [5], as it affords good protection for hyenas, and provides optimal refuges and denning sites, free from interference from humans and feral dogs [3]. The slope of the landscape was generated at 30 m resolution of Advanced Spaceborne Thermal Emission and Reflection Radiometer from global digital elevation model (ASTER GDEM; <http://asterweb.jpl.nasa.gov/gdem.asp>). Topographic features mainly terrain ruggedness, flat areas, slope (degrees), aspect were generated from elevation map using the tool Terrain Ruggedness Index (TRI) [6] in Arc Map ver. 10.2.2. The sampling area consisted of 61 % rugged terrain and 39 % flat terrain.

**Land cover type**

Due to our small sample size and to avoid complexity, we extracted four lands used and land cover types: forest; scrub forest, and riverine forest as a degraded forest; and agriculture as an anthropogenic habitat. Finally, landscape covariates such as percentage cover of the forest, degraded forest, and human settlement, and the mean and standard deviation of elevation were extracted from the 200m buffer around sampling points.

**S2 File. Datasheet for people’s perception survey in the study area of Sawai Mansingh Wildlife Sanctuary (2019-2021)**

Name of District:

Name of village/locality:

Farmer’s occupation:

Primary source of income:

Types of crops:

Livestock number:

Livestock mortality rate:

Livestock mortality season:

Livestock dumping process:

Attitude towards striped hyena:

Role of striped hyena in ecosystem:

Conflicting species:

Human-hyena conflict(yes/no):

Reason for using forest space:

**S1 Table. Classification of different land cover types present in hyena habitats in and around Sawai Mansingh Wildlife Sanctuary, Rajasthan**

| Landcover types | Descriptions |
| --- | --- |
| Forest | The land covered by ≥20% of natural vegetation such as Anogeissus pendula forest |
| Scrubland | It includes dominated by shrubs, scrub, and bushes included with grassland habitats and trees are scattered. |
| Agriculture | It includes croplands, human settlements, grazing land, and fallow lands dominated by humans. |
| Riverine habitat | It includes riparian vegetation and riverine area with gullied terrain of ravines of  Chambal River and the banks of its tributaries. |
| Water | It includes major water bodies i.e., lakes, rivers |

**S2 Table. Multicollinearity test among variables were examined using IBM SPSS Statistics V. 21.0, variables with VIF (variance inflation factor) <3 were included in the analysis**

| **Coefficients^a^** | | | | | | | | |
| --- | --- | --- | --- | --- | --- | --- | --- | --- |
| Model | | Unstandardized Coefficients | | Standardized Coefficients | t | Sig. | Collinearity Statistics | |
|  |  | B | Std. Error | Beta |  |  | Tolerance | VIF |
| 1 | (Constant) | -23.198 | 50.581 |  | -.459 | .650 |  |  |
|  | water | -3.428 | 3.375 | -.198 | -1.016 | .317 | .716 | 1.397 |
|  | human settlement | 4.956 | 8.457 | .120 | .586 | .562 | .651 | 1.536 |
|  |  |  |  |  |  |  |  |  |
|  | slope | .017 | 1.377 | .002 | .012 | .990 | .661 | 1.514 |
|  | aspect | -.015 | .043 | -.070 | -.354 | .726 | .699 | 1.430 |
|  | scrubland | -.268 | .354 | -.143 | -.756 | .455 | .758 | 1.319 |
|  | Forest cover | -.174 | .294 | -.156 | -.591 | .558 | .390 | 2.566 |
|  | riverine | .092 | .204 | .114 | .451 | .655 | .424 | 2.359 |
|  | Human presence | .028 | .107 | .061 | .261 | .795 | .494 | 2.024 |
| a. Dependent Variable: Hyena | | | | | | | | |

**S3 Table. Village wise Livestock number in the human dominated area of Sawai Mansingh Wildlife Sanctuary, Rajasthan (Based on Animal husbandry department Govt of Rajasthan 2019)**

| Village | Cattle | Buffalo | Sheep | Goat | Pig |
| --- | --- | --- | --- | --- | --- |
| Bherpura | 3 | 138 | 0 | 216 | 0 |
| Awand | 18 | 136 | 15 | 129 | 0 |
| Laxmipura | 81 | 133 | 225 | 587 | 0 |
| Lahsora | 229 | 441 | 145 | 952 | 41 |
| Ajeetpura | 27 | 127 | 44 | 406 | 0 |
| Berna | 67 | 358 | 56 | 392 | 0 |
| Badpur | 68 | 641 | 0 | 346 | 0 |
| Gandayata | 16 | 71 | 0 | 24 | 0 |
| SawaiGanj | 81 | 289 | 55 | 631 | 0 |
| Phaludi | 477 | 592 | 19 | 2569 | 81 |
| Todra | 372 | 198 | 0 | 914 | 182 |
| Jalpa Kheri | 91 | 153 | 0 | 116 | 0 |
| Hindwar | 50 | 211 | 0 | 303 | 0 |
| Dumoda | 86 | 482 | 180 | 235 | 0 |
| Kailashpuri | 31 | 838 | 50 | 263 | 0 |
| Kalakuan | 43 | 19 | 0 | 171 | 0 |
| Doloda | 171 | 375 | 0 | 455 | 0 |
| Dangarwara | 249 | 423 | 20 | 573 | 0 |
| Halonda | 143 | 237 | 80 | 482 | 0 |
| Chitara | 135 | 269 | 198 | 448 | 0 |
| Ancher | 46 | 151 | 50 | 469 | 0 |
| Hajjam Kheri | 20 | 181 | 15 | 168 | 0 |
| **Total Livestock** | 2504 | 6463 | 1152 | 10849 | 304 |

**S4 Table. RAI (Relative abundance indices of hyena and human in the study area between 2019 to 2021 along with all land use categories. RAI was calculated as RAI=E/TNx100, where E is the number of the event (photo-captures) and TN is the total number of trap nights (Palmer et al. 2018)**

| Land Use Type and Photo captures | Human | Hyena | Number of Traps | Trap Nights | RAI of Human | RAI OF Hyena |
| --- | --- | --- | --- | --- | --- | --- |
| Total | 1967 | 274 | 43 | 1462 | 134.54 | 18.74 |
| Forest | 756 | 152 | 17 | 578 | 52.15 | 26.29 |
| Scrubland | 82 | 25 | 5 | 170 | 48.23 | 14.70 |
| Agriculture | 878 | 41 | 9 | 306 | 286.92 | 13.39 |
| Riverine | 251 | 56 | 12 | 408 | 61.51 | 13.72 |

**Literature cited:**

1. Li J, Roy DP. A global analysis of Sentinel-2A, Sentinel-2B and Landsat-8 data revisit intervals and implications for terrestrial monitoring. Remote Sensing. 2017 Sep;9(9):902.
2. Strahler AH. The use of prior probabilities in maximum likelihood classification of remotely sensed data. Remote sensing of Environment. 1980 Sep 1;10(2):135-63.
3. Singh R, Qureshi Q, Sankar K, Krausman PR, Goyal SP, Nicholson KL. Population density of striped hyenas in relation to habitat in a semiarid landscape, western India. Acta Theologica. 2014 Oct 1;59(4):521-7.
4. Kruuk H. Feeding and social behaviour of the striped hyaena (Hyaena Vulgaris Desmarest). African Journal of Ecology. 1976 Jun;14(2):91-111.
5. Kushwaha SP, Roy PS. Geospatial technology for wildlife habitat evaluation. Tropical Ecology. 2002;43(1):137-50.
6. Riley SJ, DeGloria SD, Elliot R. Index that quantifies topographic heterogeneity. intermountain journal of sciences. 1999 Dec;5(1-4):23-7.
